# Enrichment of *Prevotella intermedia* in human colorectal cancer and its additive effects with *Fusobacterium nucleatum* on the malignant transformation of colorectal adenomas

**DOI:** 10.1186/s12929-022-00869-0

**Published:** 2022-10-27

**Authors:** Chia-Hui Lo, Deng-Chyang Wu, Shu-Wen Jao, Chang-Chieh Wu, Chung-Yen Lin, Chia-Hsien Chuang, Ya-Bo Lin, Chien-Hsiun Chen, Ying-Ting Chen, Jiann-Hwa Chen, Koung-Hung Hsiao, Ying-Ju Chen, Yuan-Tsong Chen, Jaw-Yuan Wang, Ling-Hui Li

**Affiliations:** 1 Institute of Biomedical Sciences, Academia Sinica, Taipei, Taiwan; 2 Division of Gastroenterology, Department of Internal Medicine, Kaohsiung Medical University Hospital, Kaohsiung, Taiwan; 3 School of Medicine, College of Medicine, Kaohsiung Medical University, Kaohsiung, Taiwan; 4 Regenerative Medicine and Cell Therapy Research Center, Kaohsiung Medical University, Kaohsiung, Taiwan; 5 Division of Colon and Rectal Surgery, Tri-Service General Hospital, Taipei, Taiwan; 6 Institute of Information Science, Academia Sinica, Taipei, Taiwan; 7 Scool of Medicine, Tzu Chi General Hospital, Taipei Branch, Taipei, Taiwan; 8 Department of Colorectal Surgery, Tzu Chi General Hospital, Taipei Branch, Taipei, Taiwan; 9 Division of Colorectal Surgery, Department of Surgery, Kaohsiung Medical University Hospital, Kaohsiung Medical University, Kaohsiung, Taiwan; 10 Graduate Institute of Clinical Medicine, College of Medicine, Kaohsiung Medical University, Kaohsiung, Taiwan; 11 Pingtung Hospital, Ministry of Health and Welfare, Pingtung, Taiwan

**Keywords:** Colorectal cancer, Microbiome, 16S rRNA sequencing, *Prevotella intermedia*, *Fusobacterium nucleatum*, Malignant transformation

## Abstract

**Background:**

Owing to the heterogeneity of microbiota among individuals and populations, only *Fusobacterium nucleatum* and *Bacteroides fragilis* have been reported to be enriched in colorectal cancer (CRC) in multiple studies. Thus, the discovery of additional bacteria contributing to CRC development in various populations can be expected. We aimed to identify bacteria associated with the progression of colorectal adenoma to carcinoma and determine the contribution of these bacteria to malignant transformation in patients of Han Chinese origin.

**Methods:**

Microbiota composition was determined through 16S rRNA V3–V4 amplicon sequencing of autologous adenocarcinomas, adenomatous polyps, and non-neoplastic colon tissue samples (referred to as “tri-part samples”) in patients with CRC. Enriched taxa in adenocarcinoma tissues were identified through pairwise comparison. The abundance of candidate bacteria was quantified through genomic quantitative polymerase chain reaction (qPCR) in tissue samples from 116 patients. Associations of candidate bacteria with clinicopathological features and genomic and genetic alterations were evaluated through odds ratio tests. Additionally, the effects of candidate bacteria on CRC cell proliferation, migration, and invasion were evaluated through the co-culture of CRC cells with bacterial cells or with conditioned media from bacteria.

**Results:**

*Prevotella intermedia* was overrepresented in adenocarcinomas compared with paired adenomatous polyps. Furthermore, co-abundance of *P. intermedia* and *F. nucleatum* was observed in tumor tissues. More notably, the coexistence of these two bacteria in adenocarcinomas was associated with lymph node involvement and distant metastasis. These two bacteria also exerted additive effects on the enhancement of the migration and invasion abilities of CRC cells. Finally, conditioned media from *P. intermedia* promoted the migration and invasion of CRC cells.

**Conclusion:**

This report is the first to demonstrate that *P. intermedia* is enriched in colorectal adenocarcinoma tissues and enhances the migration and invasion abilities of CRC cells. Moreover, *P. intermedia* and *F. nucleatum* exert additive effects on the malignant transformation of colorectal adenomas into carcinomas. These findings can be used to identify patients at a high risk of malignant transformation of colorectal adenomas or metastasis of CRC, and they can accordingly be provided optimal clinical management.

**Supplementary Information:**

The online version contains supplementary material available at 10.1186/s12929-022-00869-0.

## Background

Colorectal cancer (CRC) is one of the most prevalent malignancies worldwide [1], and colorectal adenomas (CRAs) are considered the most crucial precursor to sporadic CRC, with the adenoma–carcinoma sequence accounting for 80–85% of all CRC cases [2]. The prevalence of CRAs is estimated to be < 20% in individuals aged ≤ 50 years and 30–40% in those aged ≥ 70 years [3]. Although CRAs are considered precancerous lesions, not all adenomas progress to cancers; only 5–6% of all individuals with noncolitis CRA develop CRC [4–7]. This statistic implies a barrier to malignant transformation, and that crucial alterations of tumor cells and the microenvironment must occur in a CRA for overcoming this barrier.

Emerging evidence strongly suggests that the alteration of the gut microbiota composition contributes to CRC tumorigenesis [8–11]. Numerous studies have reported the enrichment of *B. fragilis* and *F. nucleatum* in colorectal tumors compared with that in matched normal tissues [12–18]. In a multicohort meta-analysis of the human gut microbiome from 526 fecal samples, seven CRC-enriched bacteria were identified across the analyzed populations [19]. Among these bacteria, enrichment of *B. fragilis* and *F. nucleatum* was observed across four and three cohorts, respectively, whereas enrichment of the other five bacteria, including *P. intermedia*, was noted in two of the four cohorts. Interestingly, another meta-analysis of the overlapping samples identified *P. intermedia* as the CRC-enriched microbe in American, Chinese, and French cohorts [20]. Another meta-analysis of CRC data sets involving 764 fecal genomes across multiple cohorts and populations also identified *P. intermedia* as a microbe enriched in CRC patients compared with healthy controls [21]. However, an integrative analysis of the human gut microbiome from 1267 samples collected from populations in three continents revealed country-specific gut microbial signatures [22]. Moreover, substantial regional variation of gut microbiota was observed in a study that recruited participants of Han Chinese origin living in multiple locations in Guangdong province [23]. Notably, a previous study observed considerable heterogeneity with no single operational taxonomic unit (OTU) tested being increased in all individuals with CRC [24]. Moreover, fecal microbiota only partially reflects mucosal microbiota in patients with CRC [24, 25]. These findings indicate that although some CRC-associated pathogens are common across populations, others may be population- or region-specific. Because a single bacterium cannot explain the development of all CRC cases, additional CRC-associated bacteria specific to populations or regions must be identified, which will ultimately help in the development of more effective strategies for CRC prevention or management.

To elucidate the alterations of microbes that contribute to the malignant transformation of CRA into CRC in Han Chinese residing in Taiwan, we collected autologous pairs of adenomatous polyps and adenocarcinomas and accompanying non-neoplastic colon tissue samples (referred to as “tri-part samples”) and compared the enrichment of microbes in the adenocarcinomas with that in the adenomatous polyps through 16S rRNA sequencing. The experimental results revealed that among the candidate microbes, *P. intermedia* and *F. nucleatum* were overrepresented in the adenocarcinomas. The coexistence of these two bacteria in the adenocarcinomas was associated with lymph node involvement and distant metastasis. Both bacteria enhanced cell migration and invasion and exerted an additive effect on the aforementioned biological functions, which are characteristic functions required for malignant transformation. Intriguingly, the effects of *P. intermedia* on CRC cell migration and invasion were mediated by both bacteria–cell interaction and the molecules secreted by the bacteria. This study not only reveals a new candidate pathogenic bacterium that contributes to the malignant transformation of CRA into CRC but also provide insights into the contribution of oral pathogens in the pathogenesis of CRC. Finally, our findings provide evidence that *P. intermedia* may be a population-specific bacterium contributing to CRC progression.

## Methods

### Patient recruitment

Between 2010 and 2019, 116 patients with CRC were recruited for this study. These patients underwent surgical resection for primary CRC in three collaborating hospitals: Kaohsiung Medical University Hospital, Tri-Service General Hospital, and Taipei Tzu Chi General Hospital. Of these patients, 90 patients were the same as those included in our previous study [26]. All patients were of Han Chinese origin and residing in Taiwan and had both carcinomas at any pathological TNM stage and adenomas of colons or rectums discovered during colonoscopy examination. None of the patients were diagnosed as having Crohn’s disease, ulcerative colitis, familial adenomatous polyposis, hereditary non-polyposis CRC, or hamartomatous polyposis syndrome, and none received radiation or chemotherapy prior to surgery. Patients with recurrent CRC were not included. Three autologous fresh-frozen tissues, including adenocarcinomas, paired adenomatous polyps, and paired normal colon tissues located at least 10 cm away from the malignant lesions, were collected from all patients. We also collected available clinicopathological information on cancerous tissues and polyp tissues. The histological sections of tumor samples were evaluated by experienced pathologists at each hospital. The study protocol was approved by the Institutional Review Boards of Academia Sinica and the collaborating hospitals. Informed consent was obtained from all participants before the collection of their information and samples.

### Human genomic DNA extraction and mutation detection

Human genomic DNA was isolated from the fresh-frozen tissues by using a Gentra Puregene Tissue kit (Qiagen, Valencia, CA, USA) according to the manufacturer’s instructions. Mutations were detected for 26 targeted genes in the genomic DNA of the carcinoma and paired adenomatous polyp samples from 72 patients with CRC by using the TruSight Tumor Sequencing Panel (Illumina, San Diego, CA, USA). A total of 30–300 ng of genomic DNA was used for strand-specific multiplex PCR in accordance with the manufacturer’s instructions. The quality and quantity of the amplicon-based library were measured using the Qubit and Agilent Technologies 2100 Bioanalyzer (Agilent, Santa Clara, CA, USA). Equal quantities of 16 indexed DNA libraries were pooled and sequenced on the Miseq sequencing platform (Illumina) by using a paired-end (2 × 150) sequencing run. The tri-part samples of 14 patients, including 4 in whom the targeted gene mutations were detected, were subjected to whole exome sequencing. Briefly, 3 ng of genomic DNA was utilized for whole-exome enrichment and library construction by using a TruSeq Exome Library Prep Kit (Illumina) according to the manufacturer’s instructions. Then, paired-end sequencing of 150 bases was performed using the Illumina HiSeq 2000 sequencing system. Both target sequencing and exome sequencing were conducted by the National Center for Genome Medicine at Academia Sinica, Taipei, Taiwan (http://ncgm.sinica.edu.tw). Sequencing yields of at least 10 Gb for each non-neoplastic colonic tissue sample and 20 Gb for each polyp or carcinoma sample were obtained. The data analysis is described in detail in Additional file 1.

### 16S rRNA sequencing and analysis

Genomic DNA of the tri-part samples from 93 patients with CRC was subjected to 16S rRNA sequencing, wherein 500 ng of genomic DNA was amplified with a primer pair specific to 16S V3–V4, 341F: 5′-CCTAYGGGRBGCASCAG-3′, and 806R: 5′-GGACTACNNGGGTATCTAAT-3′. Subjects with PCR products successfully amplified from more than one tissue were included in the subsequent experiments. A total of 238 samples from 86 patients were successfully amplified, and PCR products of size 450–550 bp were purified through gel extraction. The PCR amplicons were barcoded, quantified, mixed in equal amounts, and purified through gel extraction. The pooled PCR products were then used for constructing libraries by using the TruSeq DNA PCR-Free Sample Preparation Kit (Illumina). The libraries were then subjected to sequencing on a HiSeq 2500 system (Illumina) following the manufacturer’s recommendations, and 250-bp paired-end reads were generated.

All the paired-end reads were assembled using FLASH (v.1.2.11), and low-quality reads (Q score < 20) were discarded using QIIME 1.9.1. If three consecutive bases had Q scores of < 20, the read was truncated, and the resulting read was subjected to the split_libraries_fastq.py script in QIIME and retained in the data set only if it was at least 75% of the original length. Chimeric sequences were filtered using UCHIME to obtain effective tags. Samples with fewer than 15,000 effective tags were excluded from further statistical analysis. Next, OTU picking was performed by clustering effective tags with 97% sequence identity by using the USEARCH v.7 pipeline (UPARSE function). For each representative sequence, SILVA Database version 132 was used on the basis of the RDP classifier (v.2.2) algorithm to annotate taxonomy classification. Raw OTU abundance data (QIIME script single_rarefaction.py) were then rarefied to the minimum sequences of 15,000 effective tags across qualified samples through random sampling (without replacement) to avoid sampling depth bias. 16S rRNA sequencing and analysis were conducted at BIOTOOLS Co., Ltd (New Taipei City, Taiwan).

### Co-abundance group analysis

We reanalyzed the paired-end reads using mothur (v.1.39.5) for quality control and sequence alignment, and the reads were classified using the SILVA 16S rRNA database version 132 to obtain the OTU abundance table. The OTU abundance table was then used to identify microbial co-abundance groups (CAGs) by using Flemer’s method [24]. CAGs are defined as clusters of highly correlated microbes. The Euclidean distances between microbes were calculated using the Pearson correlation coefficients. The microbes were clustered using Ward’s minimum variance method with squared dissimilarities [27]. The CAGs were identified from a hierarchical cluster dendrogram with a fixed number of clusters. Vegan (Community Ecology Package, 2008) in the R environment was used for CAG identification. Pairwise comparison of the CAGs was performed using Wilcoxon tests. Significance was set as P value < 0.05.

### Bacterial culture and DNA isolation

All bacterial strains used in this study were obtained from American Type Culture Collection (ATCC, Manassas, VA, USA) and were cultured at 37 °C in an anaerobic chamber containing 10% CO_2_, 5% H_2_, and 85% N_2_. *F. nucleatum* subsp. *nucleatum* ATCC 25586 was cultured on Columbia Agar plates supplemented with 5% defibrinated sheep blood, hemin (5 µg/mL; Sigma-Aldrich, St. Louis, MO, USA), and menadione (1 µg/mL; Sigma-Aldrich). *P. intermedia* ATCC 25611 was cultured on ATCC medium 2722 supplemented with Tryptic Soy Broth/Agar, hemin (5 µg/mL), and menadione (1 µg/mL). Enterotoxigenic *B. fragilis* (Veillon and Zuber) Castellani and Chalmers (ATCC 43858) and nontoxigenic *B. fragilis* ATCC 25285 were cultured on the Anaerobic Blood Agar Plate (BD Diagnostics, Sparks, MD) or in the Brain Heart Infusion broth (Becton Dickinson, Franklin Lakes, NJ, USA) supplemented with hemin (15 µg/mL). The *Escherichia coli* strain DH5α (Invitrogen, USA) was aerobically propagated on a Luria–Bertani (LB) plate at 37 °C. Bacterial DNA was isolated from bacterial cultures by using the Gentra Puregene kit (QIAGEN, MD, USA) in accordance with the manufacturer’s instructions.

### Quantification of bacteria in tissues

Primer pairs and probes specific to human genomic sequences of the *PGT* gene and microbial genomic sequences of *P. intermedia* and *F. nucleatum* were obtained from the literature; these primer sequences are described in Additional file 2. A primer pair and probe specific to pan-bacterial 16S rRNA were purchased from Thermo Fisher Scientific (Waltham, MA, USA; Assay ID: Ba04930791_s1). qPCR was performed using 1× TaqMan Fast Advanced Master Mix (Applied Biosystems; Thermo Fisher Scientific), 180 nM of each primer, 50 nM probe, and 40 ng of human genomic DNA in a total volume of 10 μL. Assays were performed using the ViiA 7 Real-Time PCR System (Thermo Fisher Scientific) under the following thermal cycling conditions: 2 min at 50 °C, 10 min at 95 °C, and 45 cycles of 15 s at 95 °C and 1 min at 60 °C. For absolute quantification, 10× serial dilutions of the microbial genomic DNA of *P. intermedia* (ATCC 25611) and *F. nucleatum* (ATCC 25586), starting from 1 ng/μL to 10 fg/μL, were used as templates to establish a standard curve. The absolute abundance of bacteria was calculated using the interpolation or heterodyne method and the established standard curve in QuantStudio Real-Time PCR Software v.1.3 (Thermo Fisher Scientific).

### Cell culture

Human CRC cell lines were cultured as previously described [26]. HCT116 cells, HT29 cells, and HCT15 cells were cultured in McCoy’s 5A medium, Dulbecco’s Modified Eagle Medium, and RPMI 1640 medium, respectively. All cells were maintained in media supplemented with 10% heat-inactivated fetal bovine serum (FBS), 2 mM L-glutamine, 100 U/mL penicillin, and 100 µg/mL streptomycin at 37 °C in a 5% CO_2_ humidified incubator.

### Cell growth curves

Cell growth curves were determined using a hemocytometer. CRC cells were seeded in 24-well plates at a density of 2 × 10^4^ cells per well with 1 mL of antibiotic-free 10% FBS-containing complete medium. After 24 h, the cells were treated with phosphate-buffered saline (PBS)-washed bacteria at the indicated multiplicity of infection (MOI). The cells treated with PBS served as negative controls. Cell numbers were counted at 24-h intervals. In addition, the cell proliferation was measured using the Real-Time Cell Analysis (RTCA) iCELLigence System (ACEA Biosciences, San Diego, CA, USA). Briefly, CRC cells were seeded on E-plate L8 (3 × 10^4^ cells per well) containing antibiotic-free complete medium. After 24 h, the cells were treated with PBS-washed bacterial cultures at the indicated MOI. Cellular impedance was measured every 2 h for 96 h and recorded as the cell index. All experiments were performed at 37 °C in a 5% CO_2_ humidified incubator. Each experiment was performed in triplicate and repeated independently at least twice.

### Transwell migration and invasion assays

Migration and invasion assays were performed in a 24-well plate with Corning 8-µm transwell inserts, as previously described [26]. The cells were harvested and resuspended at a concentration of 1 × 10^6^/mL in antibiotic-free, serum-free medium. Next, 200 µL of the cell suspension was added to the top chamber of each 8-µm transwell insert. Bacteria pellets were then collected after centrifugation at 6000 rpm for 20 min, then washed with PBS twice, and added to the top chamber at an MOI of 100:1. To test the effect of the bacterial secretome on cell migration and invasion, 10 mL of medium from overnight-cultured bacteria was collected and centrifuged at 6000 rpm for 20 min, and the supernatant was filtered through a 0.22-µm cellulose filter (Millipore, Burlington, MA, USA). The filtered supernatant was added to the top chamber with the cells. The lower chamber contained complete medium with 10% FBS. After 18-h incubation at 37 °C, the membrane surface was fixed with paraformaldehyde and methanol, and the cells were stained with Giemsa’s staining solution and counted under a microscope. For the invasion assay, each transwell insert was precoated with 100 µL of Matrigel (Corning, NY, USA) at 0.4 µg/mL. Each experiment was performed in triplicate, and the average of the three independent experiments was calculated. To inactivate proteins in the conditioned medium, the filtered supernatant was treated with proteinase K at a final concentration of 0.1 mg/mL at 56 °C for 24 h, followed by inactivation with proteinase K at 90 °C for 10 min. The effect of the treated supernatant on cell migration was then examined. To examine the efficiency of proteinase K treatment, 30 µl of the treated and untreated conditioned media was separated using SDS-PAGE and stained using the ProteoSilver™ Silver Stain Kit (Sigma-Aldrich).

### Statistical analysis

Pairwise comparison of the relative abundance of the annotated taxa at the species level based on 16S rRNA sequencing was performed using the Wilcoxon signed-rank test. Correlations of the presence of *P. intermedia* and *F. nucleatum* with clinicopathological features and genomic or genetic alterations were determined on the basis of odds ratios (ORs). Analyses were conducted using SAS version 9.4 (SAS Institute, Cary, NC, USA). Differences in the absolute amounts of individual bacteria between the paired carcinoma samples and the polyp samples were determined using the Wilcoxon signed-rank test. Fisher’s exact test was performed to determine associations of the presence of *P. intermedia* and *F. nucleatum* in tumor tissues. The effects of the bacteria on biological functions of CRC cells were assessed using a paired *t* test. Statistical analyses were conducted using GraphPad Prism 9 (GraphPad Inc., San Diego, CA, USA). A two-tailed P value of < 0.05 was considered statistically significant.

## Results

### Relative abundance of *Prevotella intermedia* in adenocarcinomas

**Fig. 1:**
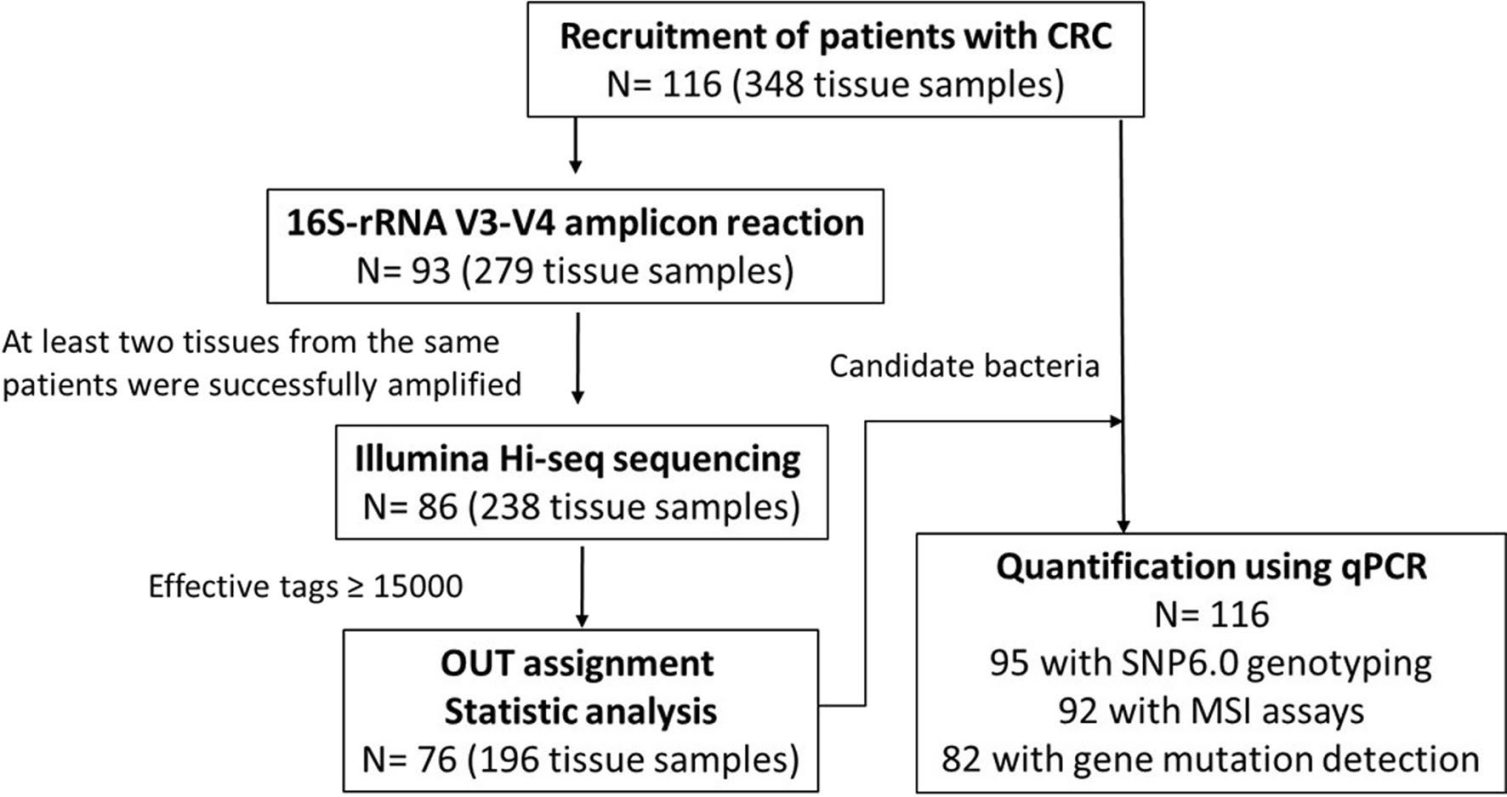
Schematic of number of patients with CRC and assays performed

The clinicopathological characteristics of the 116 patients with CRC are presented in Table 1. Genomic DNA of the tri-part samples from 93 patients was subjected to 16S-rRNA V3–V4 sequencing because this subset was available when the assay was performed (Fig. 1). Samples without successful PCR product amplification or sufficient sequencing reads were excluded from downstream analysis. Finally, a total of 196 tissue samples from 76 patients with CRC with more than 15,000 effective sequencing tags were included in a pairwise comparison to identify enriched microbes in adenocarcinoma tissues compared with those in paired adenomatous polyps and normal mucosa tissues. Only taxa annotated at the species level were analyzed (Additional file 3). The relative abundance of each bacterium in individual samples was obtained on the basis of the abundance data rarefied to 15,000 reads. Because the composition of microbiota varied considerably among individuals, we did not set a P value for the selection of enriched bacteria. Instead, we identified the top 10 overrepresented bacteria in the carcinoma samples by using the Wilcoxon signed-rank test (Table 2 and Additional file 4). Among these bacteria, *B. fragilis* was relatively enriched in carcinoma tissues compared with paired non-neoplastic colon tissues, as reported in previous studies [12, 15, 28].

**Table 1:** Characteristics of the 116 patients with CRC

Total patients	116
Gender	
Male	68 (58.6%)
Female	48 (41.4%)
Mean age, years (range)	68 (37–93)
Location of cancer	
Right	41 (35.3%)
Left	75 (64.7%)
TNM stage^a^	
Stage 0	3 (2.6%)
Stage I	21 (18.3%)
Stage II	42 (36.5%)
Stage III	40 (34.8%)
Stage IV	9 (7.8%)
Primary tumor	
T0/T1/T2	31 (26.7%)
T3/T4	85 (73.3%)
Lymph node involvement^a^	
No	66 (57.4%)
Yes	49 (42.6%)
MSI status	
MSS	80 (87.0%)
MSI-L	4 (4.3%)
MSI-H	8 (8.7%)
CIN status^b^	
Stable	14 (14.7%)
Low degree	22 (23.2%)
High degree	59 (62.1%)

^a^The regional lymph nodes of one patient could not be accessed

^b^Stable: 0 altered chromosome arms; low degree: > 1 but ≤ 10 altered chromosome arms; high degree: > 10 altered chromosome arms

**Table 2:** Top 10 enriched microbes in carcinoma tissues

taxa	TC vs. TA		taxa	TC vs. TN	
	Stat	*P* value		Stat	*P* value
[Eubacterium]_infirmum	74.0	0.0041	Campylobacter_rectus	158.5	0.0048
Streptococcus_anginosus_subsp_anginosus	262.0	0.0077	Shewanella_algae	21.0	0.0117
Aeromonas_veronii	158.0	0.0196	Streptococcus_agalactiae	83.0	0.0221
Solobacterium_moorei	166.5	0.0593	Bacteroides_fragilis	270.5	0.0303
Prevotella_intermedia	100.5	0.1153	Frisingicoccus_caecimuris	59.5	0.0336
Frisingicoccus_caecimuris	43.5	0.1325	Campylobacter_ureolyticus	32.5	0.0394
Shewanella_algae	15.0	0.1504	Culturomica_massiliensis	35.0	0.0704
Clostridium_perfringens	41.5	0.1533	Streptococcus_anginosus_subsp_anginosus	173.5	0.0748
Lactococcus_garvieae_subsp_garvieae	13.0	0.1563	Lactobacillus_mucosae	13.0	0.0859
Streptococcus_dysgalactiae_subsp_ equisimilis_GGS_124	17.5	0.1812	Bifidobacterium_bifidum	30.0	0.0872

*TC* carcinoma tissue, *TA* polyp tissue, *TN* non-neoplastic tissue

We were particularly interested in bacteria that were relatively enriched in adenocarcinomas compared with paired adenomatous polyps because these bacteria may be involved in the malignant transformation of adenomas into adenocarcinomas. Among the top 10 bacteria, the present study focused on *P. intermedia*. *P. intermedia* was reported to be enriched in the fecal samples of patients with CRC compared with healthy controls in the Austrian, Chinese, American, and French populations, but not in German population [19, 20]. However, no study has reported the abundance of *P. intermedia* in colorectal carcinoma tissues.

### Co-abundance of *Prevotella intermedia* and *Fusobacterium nucleatum* in carcinoma tissues compared with paired polyp tissues validated through qPCR

**Fig. 2:**
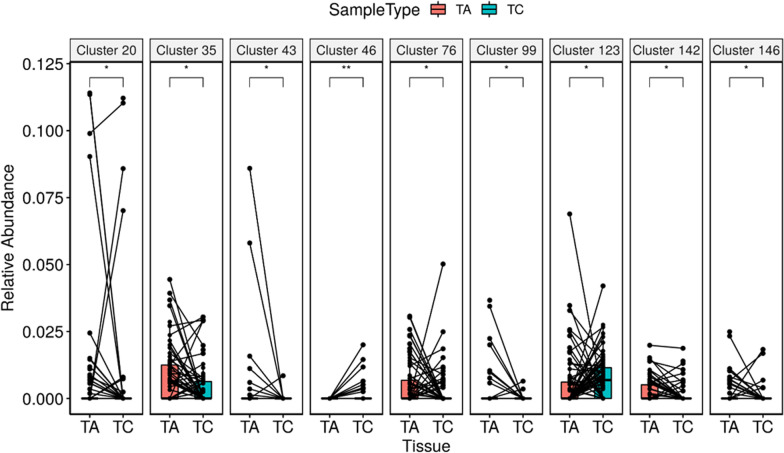
Differentially abundant co-abundance groups (CAGs) of cancer and polyp tissues. The differentially abundant CAGs of cancer tissues (TC) and polyp tissues (TA) are displayed in box plots overlaid with paired dot plots. *P value < 0.05; **P value < 0.01.

To verify the 16S rRNA sequencing result, we performed genomic qPCR to measure the absolute amount of *P. intermedia* in adenocarcinoma tissues and paired polyp tissues from the 76 patients with CRC (Fig. 1). Forty more patients with CRC were included in the qPCR assays to increase the statistical power. Although *F. nucleatum* was not identified in the sequencing analysis because of the low taxonomical resolution of the V3-V4 amplicon, CAG analysis of the OTUs revealed that *Prevotella* and *Fusobacterium* were co-abundant in cancer tissues compared with polyp tissues (Fig. 2 and Additional file 5). CAG Cluster 46 comprised f_Prevotellaceae; g_Prevotella, f_Peptococcaceae; g_SCADC1-2-3, f_Fusobacteriaceae; g_Fusobacterium, Fusobacteriales, and f_Methylomonaceae; g_Methylobacter and exhibited higher abundance in adenocarcinomas than in adenomatous polyps (P value = 0.0046, Fig. 2) and normal mucosa tissues (P value = 0.0146, Additional file 5). Therefore, we also measured the quantity of *F. nucleatum* through qPCR.

**Fig. 3:**
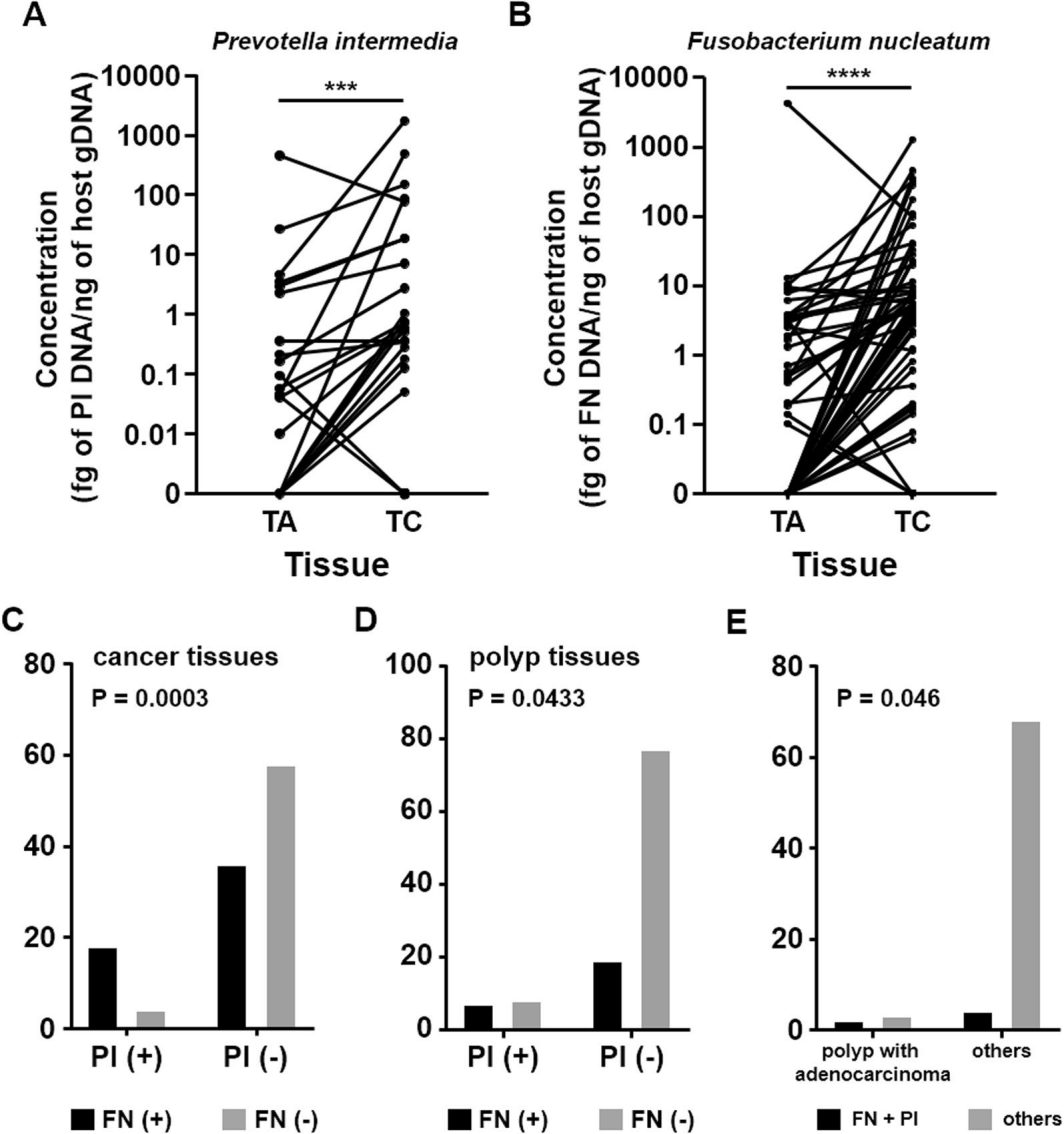
Co-abundance of *Prevotella intermedia* and *Fusobacterium nucleatum* increases from polyps to paired carcinoma tissues. The absolute abundance of **A** *Prevotella intermedia* and **B** *Fusobacterium nucleatum* increases from polyps (TA) to paired carcinomas (TC). The quantification of bacteria is presented as the amount of microbial genomic DNA per ng of genomic DNA isolated from corresponding tissues. *TA* adenomatous polyp, *TC* carcinoma tissue. Tissue samples from the same patients with CRC are linked by lines. ***P value < 0.001; ****P value < 0.0001. The numbers of tissues with the presence or absence of *Prevotella intermedia* (PI) and *Fusobacterium nucleatum* (FN) in **C** cancer tissues and **D** polyp tissues are presented. **E** The coexistence of *Prevotella intermedia* (PI) and *Fusobacterium nucleatum* (FN) is predominant in polyps with adenocarcinoma lesions

With the threshold set at cycle 45, *P. intermedia* and *F. nucleatum* were detected in at least one of the tri-part tissue samples of 25 patients (21.6%) and 60 patients (51.7%), respectively. More specifically, *P. intermedia* and *F. nucleatum* were detected in the carcinoma tissues of 22 (19.0%) and 54 (46.6%) patients, respectively. According to the available information on the histological types of polyps, five patients had adenocarcinoma lesions within their adenoma tissues and were excluded from the paired tissue comparison. The absolute abundance of *P. intermedia* in carcinoma tissues was significantly higher than that in paired polyp tissues (P value = 0.0003, Fig. 3A). A similar result was obtained for *F. nucleatum* (P value < 0.0001, Fig. 3B). The qPCR result was consistent with that of the 16S rRNA sequencing data enrichment analysis. Notably, this result indicated that *P. intermedia* and *F. nucleatum* coexisted in both the carcinoma tissues (Fig. 3C, P value = 0.0003, Pearson’s correlation coefficient = 0.85) and polyp tissues (Fig. 3D, P value = 0.0433, Pearson’s correlation coefficient = 0.99) of the patients with CRC. Moreover, the coexistence of these two bacteria was significantly associated with polyps with adenocarcinoma lesions (P value = 0.046, Fig. 3E). In addition, of the five polyps with higher abundance of *P. intermedia* compared with the paired adenocarcinomas, two had carcinoma lesions within adenoma tissues. These findings strongly suggest that *P. intermedia* is associated with the malignant transformation of CRA into CRC.

### Associations of bacteria with clinicopathological features and genomic alterations

Next, we determined the associations of the presence of the two bacteria in carcinoma samples with clinicopathological features (Table 3). The abundance of a bacterium was categorized as a dichotomous variable (present vs. absent), and ORs were calculated. The coexistence of *P. intermedia* and *F. nucleatum* was significantly associated with distant metastasis (OR = 5.31, 95% confidence interval [CI] = 1.27–22.2). The association of the presence of *P. intermedia* or *F. nucleatum* alone with distant metastasis was marginally significant (OR = 3.96, 95% CI = 0.97–16.19 and OR = 4.47, 95% CI = 0.89–22.5, respectively). Although not statistically significant, the coexistence of these two bacteria were associated with the TNM stage (OR = 2.89, 95% CI = 0.99–8.48) and lymph node involvement (OR = 2.89, 95% CI = 0.99–8.48). The association of the presence of *F. nucleatum* with the TNM stage and lymph node involvement was statistically significant (OR = 2.27, 95% CI = 1.08–4.8). Gender, tumor location, or primary tumor was not associated with the presence of either bacterium.

**Table 3:** Association of bacteria with clinicopathological features and genomic alterations

		*Prevotella intermedia*			*Fusobacterium nucleatum*			*P. intermedia* + *F. nucleatum*		
		Presence	Absence	OR (95% CI)	Presence	Absence	OR (95% CI)	Co-existence	Others	OR (95% CI)
Gender	Male	14	54	1.30 (0.50, 3.39)	34	34	1.40 (0.66, 2.95)	13	55	2.03 (0.67, 6.14)
	Female	8	40		20	28		5	43	
Location	Left	14	61	0.95 (0.36, 2.49)	32	43	0.64 (0.30, 1.38)	14	61	2.12 (0.65, 6.94)
	Right	8	33		22	19		4	37	
TNM stage	Late (III/IV)	12	37	2.05 (0.79, 5.35)	28	21	2.19 (1.03, 4.64)	11	38	2.89 (0.99, 8.48)
	Early (0/I/II)	9	57		25	41		6	60	
Primary tumor	T3/T4	16	69	0.97 (0.34, 2.77)	40	45	1.08 (0.47, 2.46)	14	71	1.33 (0.40, 4.40)
	T0/T1/T2	6	25		14	17		4	27	
Lymph node	Yes	12	37	2.05 (0.79, 5.35)	28	21	2.19 (1.03, 4.64)	11	38	2.89 (0.99, 8.48)
Involvement	No	9	57		25	41		6	60	
Distant metastasis	Yes	4	5	3.96 (0.97,16.19)	7	2	4.47 (0.89, 22.51)	4	5	5.31 (1.27, 22.20)
	No	18	89		47	60		14	93	
CIN status	High	13	46	1.75 (0.57, 5.41)	29	30	0.97 (0.42, 2.21)	12	47	2.04 (0.60, 6.90)
	Low or stable	5	31		18	18		4	32	
Chr20 amplification	Yes	12	54	0.85 (0.29, 2.55)	30	36	0.59 (0.24, 1.42)	11	55	0.96 (0.30, 3.06)
	No	6	23		17	12		5	24	
Chr13q amplification	Yes	12	37	2.16 (0.74, 6.35)	27	22	1.60 (0.71, 3.59)	11	38	2.37 (0.75, 7.46)
	No	6	40		20	26		5	41	
MSI status	High	3	5	2.76 (0.59, 12.83)	6	2	3.15 (0.60, 16.49)	3	5	3.28 (0.70, 15.42)
	Low or stable	15	69		41	43		13	71	
APC mutation	Yes	9	35	0.96 (0.33, 2.81)	19	25	0.50 (0.21, 1.20)	7	37	0.71 (0.23, 2.18)
	No	8	30		23	15		8	30	
KRAS mutation	Yes	7	24	1.20 (0.40, 3.55)	17	14	1.26 (0.52, 3.09)	5	26	0.79 (0.24, 2.57)
	No	10	41		25	26		10	41	
TP53 mutation	Yes	13	42	1.78 (0.52, 6.09)	28	27	0.96 (0.38, 2.42)	13	42	3.87 (0.81, 18.58)
	No	4	23		14	13		2	25	

*OR* odds ratio, *CI* confidence interval, *CIN* chromosomal instability, *MSI* microsatellite instability

We further examined the associations of the presence of the two bacteria in the carcinoma samples with genomic and genetic alterations wherever the data were available. Our previous study demonstrated that Chr20q amplification and Chr13q amplification were the characteristic genomic alterations involved in the malignant transformation of CRA into CRC [26]. *APC*, *KRAS*, and *TP53* genes are the most frequently mutated genes in CRC. Therefore, we examined the association of the two bacteria with these mutational events, and the results revealed that the coexistence of *P. intermedia* and *F. nucleatum* was associated with the *TP53* mutation; however, this association did not reach statistical significance (OR = 3.87, 95% CI = 0.81–18.58). No other associations were observed between other mutational events and either of the two bacteria.

### Effects of *Prevotella intermedia* on CRC cell growth in various cell lines and at different MOIs

**Fig. 4:**
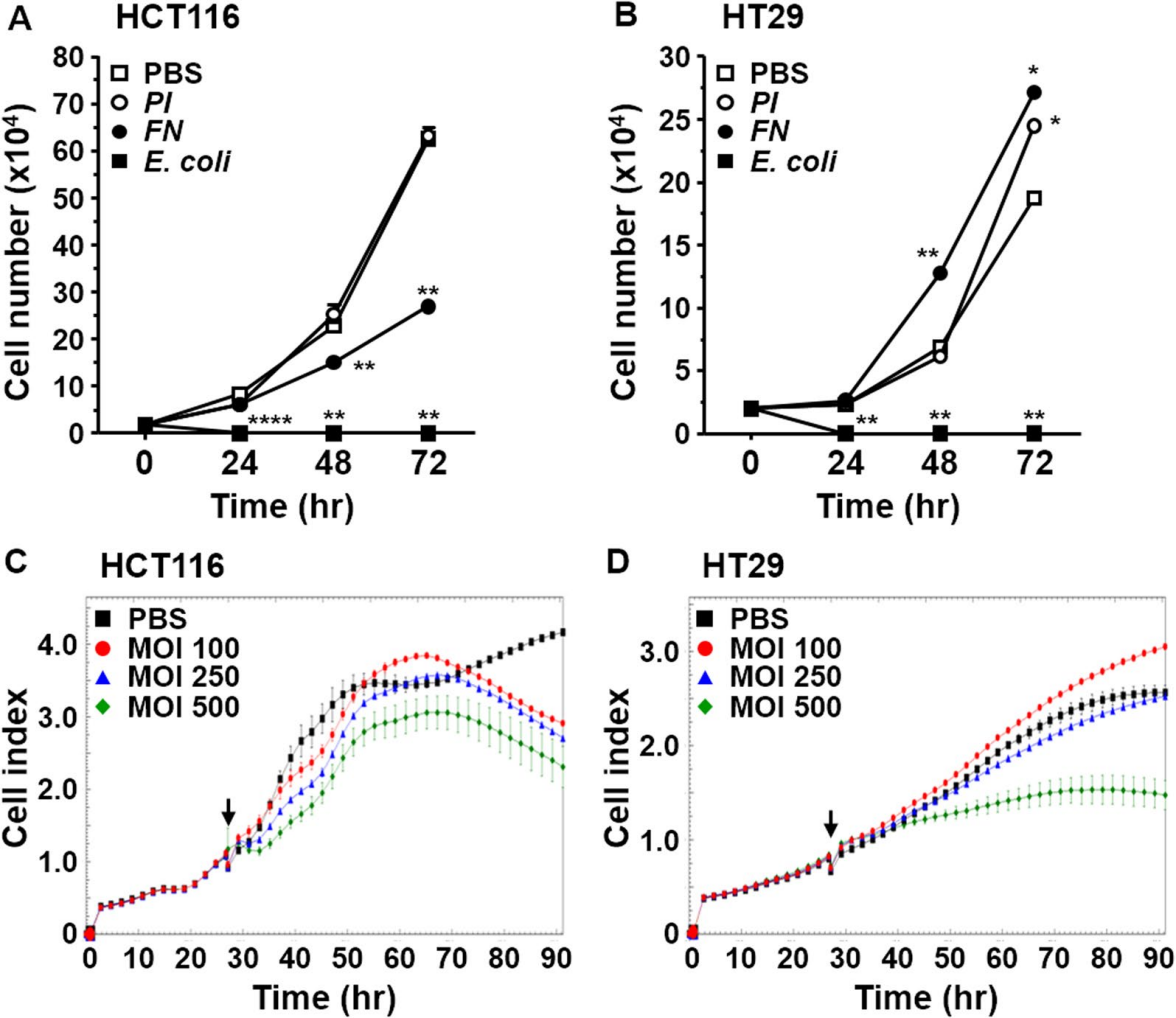
Effects of *Prevotella intermedia* on cell growth varied across cell lines and MOIs. **A** HCT116 cells and **B** HT29 cells were treated with *Prevotella intermedia* (PI), *Fusobacterium nucleatum* (FN), or *E. coli DH5a* at an MOI of 100 on day 0. Cell growth curves were measured using a hemocytometer. Cells treated with PBS served as controls. **C** HCT116 cells and **D** HT29 cells were co-cultured with *Prevotella intermedia* at various MOIs, and cell growth was measured using the RTCA iCELLigence System. The arrowhead indicates the time when the bacteria were added to the cell culture. *P value < 0.05; **P value < 0.01; ****P value < 0.0001

To investigate whether *P. intermedia* is involved in CRC development, we first examined whether the growth of CRC cells was influenced by the direct interaction between bacteria and cancer cells. Compared with PBS-treated cells, the growth of HCT116 cells was not affected by *P. intermedia* at an MOI of 100 (Fig. 4A). The growth of HT29 cells increased after 48–72-h incubation with *P. intermedia* at an MOI of 100 (Fig. 4B). We co-cultured *F. nucleatum* with CRC cells as a control. Unexpectedly, the growth of HCT116 cells was suppressed, whereas that of HT29 cells was promoted after 24-h incubation at an MOI of 100 (Fig. 4A, B). *Escherichia coli* DH5α treatment resulted in cell damage in both cell lines after 24-h incubation at an MOI of 100 (Fig. 4A, B). We then employed cell index measurement to examine the growth of CRC cells incubated with *P. intermedia* at various MOIs. The cell index of HCT116 cells was similar between the PBS control group and the groups treated with bacteria at an MOI of 100, except after 40-h incubation. However, the cell index of HCT116 cells was lower after incubation with *P. intermedia* at an MOI of 250 or 500 compared with that of the cells treated with PBS only (Fig. 4C). In HT29 cells, the cell index increased at an MOI of 100 but decreased at an MOI of 250 or 500 (Fig. 4D).

### *Prevotella intermedia* and *Fusobacterium nucleatum* additively enhanced the migration and invasion of CRC cells

**Fig. 5:**
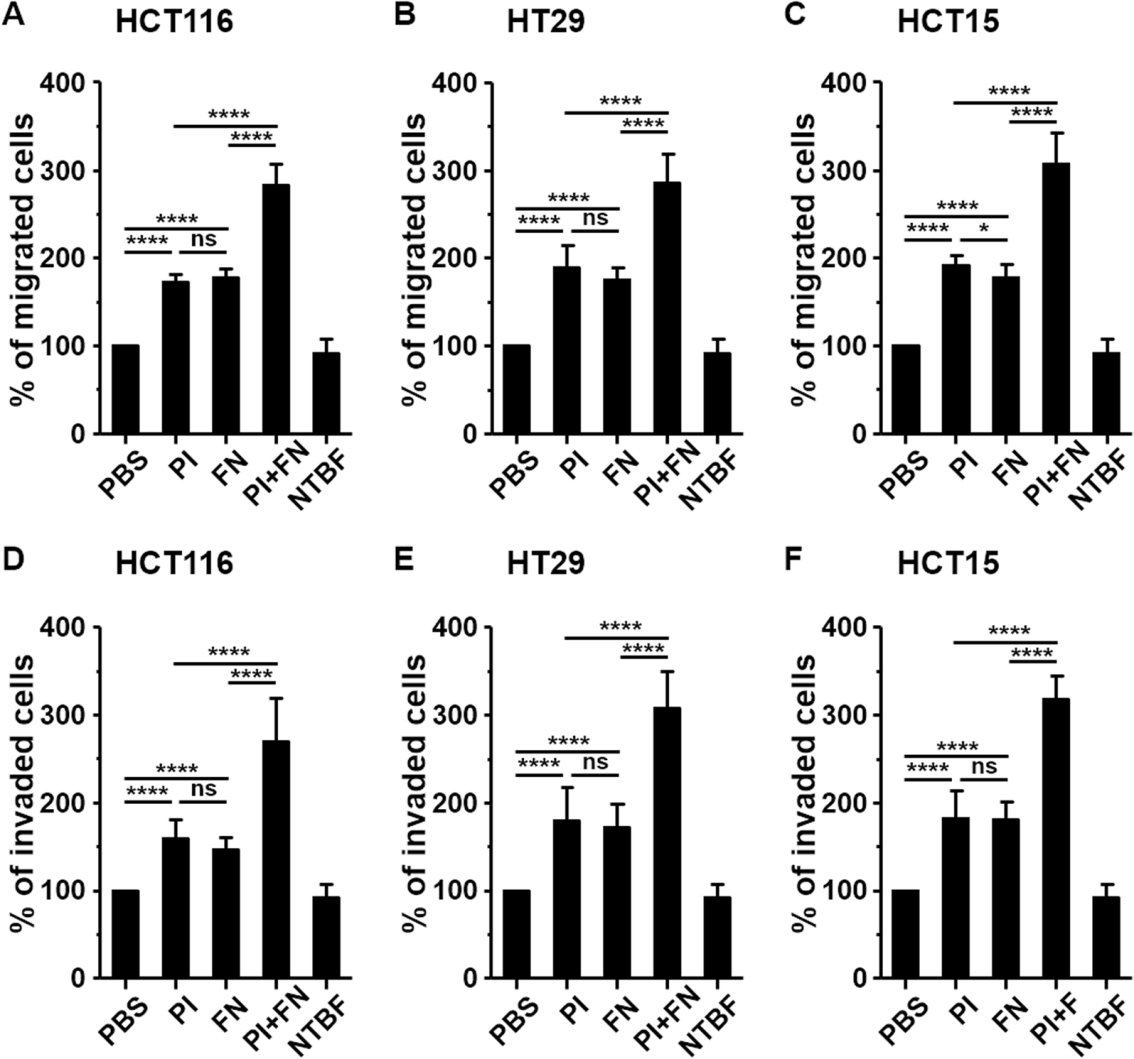
*Prevotella intermedia* and *Fusobacterium nucleatum* additively enhances CRC cell migration and invasion. **A**–**C** Migration and **D**–**F** invasion assays were performed on CRC cells cultured with *Prevotella intermedia* (PI), *Fusobacterium nucleatum* (FN), PI plus FN (PI + FN), or nontoxigenic *B. fragilis* (NTBF) at an MOI of 100. Cells treated with PBS served as untreated controls, and NTBF served as a negative control. The relative percentage of migrated and invaded cells is presented as mean ± standard deviation. Data were obtained from three independent experiments. *P value < 0.05; ****P value < 0.0001; *ns* not significant

Migration and invasion are hallmarks of malignant transformation. We investigated whether *P. intermedia* influenced the migration and invasion abilities of CRC cells. Compared with PBS treatment and nontoxigenic *B. fragilis*, *P. intermedia* significantly promoted the migration of HCT116, HT29, and HCT15 cells at an MOI of 100 (Fig. 5A–C). Moreover, *P. intermedia* substantially enhanced cell invasion in the three CRC cell types (Fig. 5D–F). To examine whether the ability of *P. intermedia* to promote cell migration and invasion was reinforced by an increased number of bacteria, we incubated CRC cells with bacteria at a higher MOI; the results revealed that the percentages of migrated and invaded cells were similar at MOIs of 100 and 250, suggesting no dose-dependent effect (Additional file 6).

Consistent with the findings of other studies [29, 30], *F. nucleatum* promoted the migration and invasion of the three CRC cell lines at an MOI of 100. Because *P. intermedia* and *F. nucleatum* were observed to coexist in human carcinoma tissues, we further investigated whether *P. intermedia* in combination with *F. nucleatum* exerted a synergistic effect on cell migration or invasion. We observed that both migration and invasion were significantly promoted in CRC cells co-cultured with the combination of the two pathogens compared with those co-cultured with either pathogen alone. These findings revealed an additive effect of *P. intermedia* and *F. nucleatum* on the promotion of migration and invasion of CRC cells.

### Secretome of *Prevotella intermedia* enhanced migration and invasion of CRC cells

**Fig. 6:**
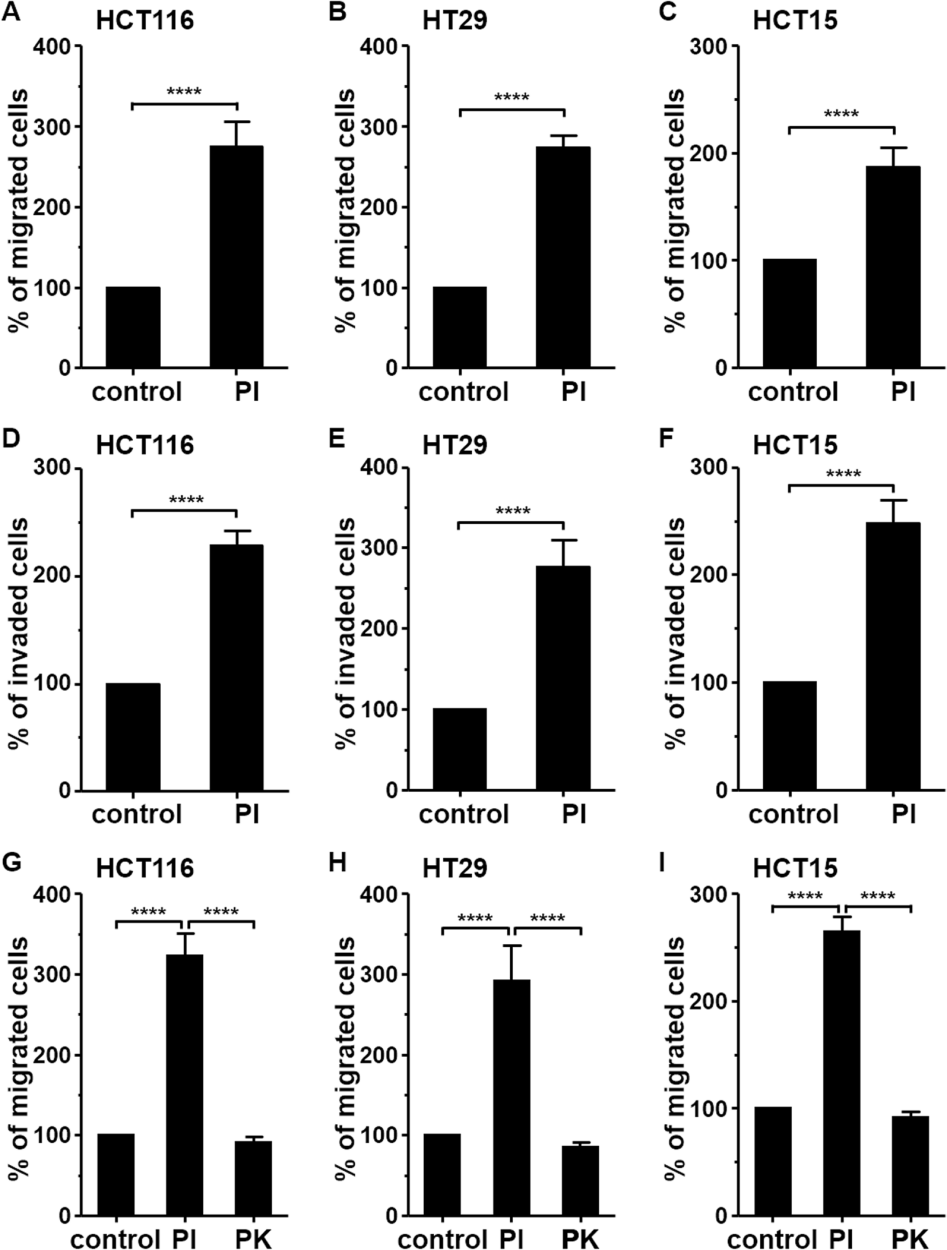
Conditioned medium from *Prevotella intermedia* enhances CRC cell migration and invasion. CRC cells were incubated in the conditioned medium from *Prevotella intermedia* in the top chamber for migration (**A**–**C**) and invasion (**D**–**F**) assays. **G**–**I** Effect of proteinase K-treated conditioned medium from *Prevotella intermedia* on cell migration. The relative percentages of the migrated and invaded cells are presented as mean ± standard deviation. Data were obtained from three independent experiments. ****P value < 0.0001

Few studies have explored the cancer pathogenicity of *P. intermedia*. Therefore, the present study investigated whether *P. intermedia* stimulate the migration or invasion of CRC cells through its secretory factors or through direct contact with cells. Conditioned medium from *P. intermedia* significantly enhanced the migration and invasion of HCT116, HT29, and HCT15 cells compared with growth medium alone (Fig. 6A–F). However, when the conditioned medium was treated with proteinase K, the effects were eliminated (Fig. 6G–I). Moreover, the secreted proteins were destroyed, which was verified through gel electrophoresis (Additional file 7). Collectively, these results indicate that *P. intermedia* can exert its effects on cell migration and invasion through secreted proteins as well as through direct cell contact.

## Discussion

Because of the low resolution of the taxonomy assignment based on 16S rRNA V3-V4 sequencing, most studies have reported microbiota dysbiosis of CRC at the genus level. For example, Liu et al. reported that the genus *Prevotella* was more abundant in biopsy samples from patients with CRC than in those from patients with CRA in one of the two cohorts from two geographically separated cities in China [31]. However, no information at the species level was available. In addition, because of the heterogeneity of microbiota among individuals and populations, only *F. nucleatum* and *B. fragilis* have been clearly identified as species enriched in fecal samples and tumor samples taken from patients with CRC across studies. Although *P. intermedia* has been identified as a CRC-enriched microbe, this finding was based on a metagenomic analysis of fecal samples; no study has demonstrated the presence of this bacterium in tumor tissues. The present study demonstrated that *P. intermedia* and *F. nucleatum* were enriched in carcinoma tissues compared with autologous adenomatous polyp tissues. Notably, this finding was confirmed by the absolute quantification of these two bacteria in 116 patients with CRC. To the best of our knowledge, the present study included the largest collection of samples of paired colorectal carcinoma and polyp tissues to date. The effects of *P. intermedia* on the malignant properties of CRC cells strongly suggest that *P. intermedia* is a pathogenic microbe that contributes to the malignant transformation of CRA into CRC. Intriguingly, the results revealed that the coexistence of *P. intermedia* and *F. nucleatum* was associated with polyp tissues with carcinoma lesions. These findings support the pathogenic role of the combination of *P. intermedia* and *F. nucleatum* in the progression of CRA to CRC.

Our finding of the coexistence of *P. intermedia* and *F. nucleatum* in cancerous colon tissue is consistent with the previous findings that *Fusobacterium* cooccurs with gram-negative anaerobic oral bacteria [13, 32, 33]. In particular, the co-abundance of the genera *Fusobacterium* and *Prevotella* was observed in a subset of CRC samples in another study [24]. Both *F. nucleatum* and *P. intermedia* are oral bacteria and are implicated in the pathogenesis of periodontitis. One study involving the in vitro co-culture of *F. nucleatum* and *P. intermedia* demonstrated that these two bacteria were coaggregated [34]. Moreover, in other studies, a co-culture of *F. nucleatum* and *P. intermedia* significantly enhanced biofilm formation compared with the single culture of *F. nucleatum* [34, 35]. These two bacteria may be a part of a strong symbiotic network that originates in oral cavities and colonizes the gut to promote malignant transformation. Avuthu and Guda performed a meta-analysis of existing metagenomic data sets from diverse geographic regions to identify a set of global biomarkers for CRC [20]. The co-occurrence network analysis of fecal microbes revealed that a cluster of oral microbes including *F. nucleatum* and *P. intermedia* was associated with CRC. This result is in line with our findings for tumor tissues. In the future, we will employ murine models to elucidate the potential oncogenic role of *P. intermedia* and how it cooperates with *F. nucleatum* to promote CRC metastasis. Moreover, whether *P. intermedia,* in addition to *F. nucleatum*, in stool samples can serve as a diagnostic biomarker for CRC in Chinese population will be evaluated. Our result of the co-abundance of these two bacteria in human colorectal carcinoma tissues supports that the alteration of the ecosystem consisting of the oral microbiome is involved in CRC development [24, 33]. Because the coexistence of *P. intermedia* and *F. nucleatum* was found in only a subset of patients with CRC in this study, we suspect that other oral pathogens may also coexist with *F. nucleatum.* Therefore, shot-gun metagenomic sequencing of tissue samples should be used in the future to demonstrate the other bacterial species coexisting with *F. nucleatum*. Moreover, using the metadata collected from CRC patients, we will implement a model in explainable artificial intelligence (AI) to predict the stages of CRC based on the gut microbiota and selected features from metadata. This will help identify the most important microbiota community and features, thereby enabling the development of more sensitive models to detect CRC at the early stage.

The *TP53* mutation is a critical genetic event contributing to the malignant transformation of CRA into CRC with chromosomal instability. In this study, the trend of association of the coexistence of *P. intermedia* and *F. nucleatum* with the *TP53* mutation, suggesting that these two bacteria may contribute to the malignant transformation of CRA into CRC through genotoxicity. The most widely known example of genotoxin-induced *TP53* mutation is the synergistic effect of aflatoxin produced by fungi and chronic hepatitis B virus infection in hepatocellular carcinoma [36]. Similarly, we propose that along with the invasion of CRA tissues with *F. nucleatum*, the to-be-identified secreted proteins of *P. intermedia* may induce host gene mutations. The clonal expansion of cells with *TP53* mutations and their relatively high ability of both migration and invasion render benign tumors malignant. Studies have reported the associations of the number of *F. nucleatum* with high microsatellite instability (MSI) cancer tissue [37–39]; however, no such association was observed in the present study. This inconsistency is likely due to the small number of MSI-high samples included in this study. Additionally, the associations of *F. nucleatum* with right-sided tumors, advanced stage tumors, and *BRAF*-mutated tumors were reported in a Brazilian cohort [40]. The present study observed an association of *F. nucleatum* with the high TNM stage, but not with right-colon cancers or *BRAF* mutations in our cohort. Similarly, no association between *F. nucleatum* abundance and *KRAS*-mutation-positive CRC was observed in a Chinese cohort [39], which is consistent with our finding. Therefore, we speculate that the associations of the abundance of *F. nucleatum* with clinicopathological and molecular features may be population-specific. Although studies with more diverse populations and larger sample sizes are required to determine whether population-specific bacteria are responsible for CRC progression and are associated with clinicopathological characteristics, our results strongly suggest that *P. intermedia* and *F. nucleatum* are critical pathogens for CRC development in individuals of Han Chinese origin. Moreover, our recent study demonstrated that *F. nucleatum* is closely associated with treatment outcomes in patients with metastatic CRC receiving chemotherapy and targeted therapy [41].

To investigate the potential pathogenic role of *P. intermedia* in CRC progression, we conducted a series of experiments involving the incubation of CRC cells with bacterial cells or conditioned medium from bacteria. The results revealed that *P. intermedia* significantly enhanced cell migration and invasion, and these effects were consistently observed in the three CRC cell lines. Moreover, the combination of *P. intermedia* and *F. nucleatum* exerted additive effects on the promotion of CRC cell migration and invasion. The findings regarding the co-abundance of these two bacteria in carcinoma tissues compared with paired polyp tissues and the association of the coexistence of these two bacteria with lymph node involvement and distant metastasis strongly suggest that *P. intermedia* is involved in the malignant transformation of CRA and metastasis in combination with *F. nucleatum*. In a recent study, Ternes and colleagues reported that gut microbial metabolite formate is a key player in CRC progression through the activation of AhR signaling [42]. The microbiome-derived formate induces cancer stemness, promotes colon cancer cell invasion, and ultimately induces metastatic dissemination. The authors suggest that *F. nucleatum* is one of the microbes enriched in CRC tissues that produce formate at the tumor site, and that bacterial species other than *F. nucleatum* may also contribute to formate production. Intriguingly, the bacterium *P. intermedia* contains a series of enzymes essential for formate formation, and it can metabolize glucose into formate [43]. The additive effect of *P. intermedia* and *F. nucleatum* on the migration and invasion of CRC cells may be explained by the increasing concentration of formate. However, confirming whether tumor-associated *P. intermedia* contributes to formate formation in patients with CRC is difficult because it coexists with *Fusobacteria nucleatum* in our study cohort. Nevertheless, based on the association of the coexistence of these two bacteria with malignant transformation and metastasis of CRC and considering that formate may function as a major oncometabolite through the activation of AhR signaling, AhR inhibitors are of potential therapeutic interest in patients with CRC with both the bacteria detected [44]. We propose that the follow-up visit intervals for patients with both bacteria present in adenoma tissues should be shortened. Moreover, we propose that the formate levels of CRC patients with the presence of these two bacteria in the cancerous tissues should be detected to monitor disease progression. Additionally, AhR inhibitors may be administered to this subgroup of patients to prevent metastasis when the drugs are available.

In this study, conditioned medium from *P. intermedia* exerted a more prominent effect on cell migration and invasion compared with the bacterial cells. The virulence factors mediating the contribution of *P. intermedia* to malignant transformation may be secreted proteins given that the proteinase K treatment of the conditioned medium eliminated these biological activities. In a recent report, an analysis of the functional protein association network of *P. intermedia* secretomes revealed that carbohydrate metabolism constitutes one of the three major groups in the network [45]. Among the components of the sugar metabolism network, glucose-6-phosphate isomerase (GPI), also known as autocrine motility factor (AMF), was identified. AMF secreted from tumor cells exhibits non-canonical function and signaling through its receptor gp78 and drives epithelial–mesenchymal transition (EMT) in several cancers [46–48]. AMF has also been demonstrated to stimulate invasion and metastasis in various cancers [47, 49, 50]. We speculate that AMF is one of the molecules secreted by *P. intermedia*, and that it is responsible for promoting CRC cell motility and ultimately metastasis. Future studies should attempt to detect AMF in colorectal cancer tissues with the presence of *P. intermedia*. Because AMF-induced metastasis occurs through the non-canonical pathway, targeting AMF to inhibit its contribution to metastasis without affecting its physiological functions is feasible. Efforts toward developing monoclonal antibodies against AMF have demonstrated encouraging results [51, 52]. Moreover, studies including more clinical samples and applying animal models [38] for the identification of more secreted molecules and the mechanisms by which they promote CRC progression may assist in the prevention of malignant transformation and metastasis of CRC. Together, these results suggest that *P. intermedia*—both the bacterial cells and their secreted components—may play a pathogenic role in the malignant transformation of CRA into carcinoma.

Cell proliferation is another key feature of tumorigenesis. In this study, we demonstrated various effects of *P. intermedia* on CRC cell growth. Specifically, *P. intermedia* selectively promoted the growth of HT29 cells, but not that of HCT116 cells, at an MOI of 100. This effect may partially be explained by the varying gene mutations in different cell lines, which are associated with different signaling responses and metabolic pathways. To the best of our knowledge, the effect of *P. intermedia* on cancer cell growth has not been reported previously. However, one study demonstrated that some bacterial cells as well as their secretomes were associated with either the enhancement or inhibition of cancer cell growth [53]. Studies have demonstrated that *F. nucleatum* can increase the proliferation of HCT116, HT29, and SW480 CRC cells [16, 30] and can promote cell growth through Toll-like Receptor 4 signaling and microRNA-21 upregulation through its binding to FadA. However, we observed that *F. nucleatum* inhibited the growth of HCT116 cells but enhanced the growth of HT29 cells at an MOI of 100. Moreover, after incubation with bacteria at higher MOIs of up to 1000, the growth of CRC cells was arrested, and the cells were damaged (Additional file 8). Therefore, differences in MOI or the experimental materials cannot explain the contrasting results, because we used the same bacterial strain (ATCC 25586), culture medium, and assay techniques. However, differences in co-culture conditions may exist among the study groups. To sustain cell growth, we established the co-culture in an aerobic chamber unsuitable for anaerobic bacterial growth. Notably, one recent report revealed that *Fusobacteriaceae* significantly inhibited CRC cell growth [53], suggesting that the bacterial characteristics must be elucidated to further explain the inconsistent results. Notably, we observed no association of the presence of *P. intermedia* or *F. nucleatum* with the size or extent of the primary tumor (T0/T1/T2 vs. T3/T4). This finding is consistent with the result of the cell proliferation assay in this study.

This study has several limitations. First, the study patients were of Han Chinese origin and were residing in Taiwan. Therefore, it requires further investigation to determine the generalization of the results to CRC patients of Han Chinese origin residing in other locations and to other populations. Second, no independent cohort was available in this study for replication. Autologous adenomas and adenocarcinomas from additional patients with CRC should be collected. Finally, the low resolution of 16S rRNA sequencing is an obstacle to the detection of bacteria at species level. Some important bacterial species responsible for malignant transformation may not be identified by 16S rRNA sequencing. Shotgun metagenomic sequencing coupled with complete depletion of host DNA from tissue samples will provide a comprehensive list of microbes that contribute to malignant transformation of CRA at the species and even strain levels.

## Conclusion

This study demonstrated that *P. intermedia* plays a crucial role in the promotion of both migration and invasion of CRC cells. Furthermore, the coexistence of *P. intermedia* and *F. nucleatum* exerted an additive effect on the migration and invasion of CRC cells and was associated with both lymph node metastasis and distant metastasis. Therefore, patients with these two bacteria present in their precancerous tissues may be at a relatively high risk of developing colorectal carcinomas, and patients with these two bacteria present in cancerous tissues may be at a relatively high risk of metastasis. Both these conditions necessitate the increased monitoring of patients. In addition, the identification of the pathogenic molecules from *P. intermedia* and the response molecules from host cells as well as the relevant mechanical pathways warrants further investigation. The discovery of these mechanisms can help establish optimal strategies for preventing the malignant transformation of CRA and metastasis of CRC.

## Supplementary Information

**Additional file 1.** Details of data analysis of tumor-gene panel sequencing and exome sequencing.

**Additional file 2.** Primer and probe sequences specific to species for bacterial DNA quantification. Primer and probe sequences specific to species for bacterial DNA quantification.

**Additional file 3.** Relative abundance of taxa at species level. Relative abundance of taxa annotated at species level of 196 tissue samples from 76 CRC patients.

**Additional file 4.** Wilcoxon signed-rank test. Wilcoxon signed-rank test of the relative abundance of species in pairwise comparison.

**Additional file 5.** Genera included in co-abundance groups with significant alteration. T-test results and genera of the co-abundance groups with significant alteration among cancer tissues, polyp tissues, and non-neoplastic colon tissues.

**Additional file 6: Figure S1.** Migration and invasion of CRC cells incubated with *Prevotella intermedia* at various MOIs.

**Additional file 7: Figure S2.** Silver staining of conditioned medium collected from *Prevotella intermedia*.

**Additional file 8: Figure S3.** Growth of CRC cells incubated with *Prevotella intermedia* at various MOIs.

## Data Availability

All data generated or analyzed during this study are included in this published article and its Additional files.

## Abbreviations

CRC: Colorectal carcinoma
qPCR: Quantitative polymerase chain reaction
*P. intermedia*: *Prevotella* *intermedia*
*F. **nucleatum*: *Fusobacterium* *nucleatum*
CRA: Colorectal adenoma
*B. fragilis*: *Bacteroides fragilis*
OUT: Operational taxonomic unit
CAGs: Co-abundance groups
OR: Odds ratio
CI: Confidence interval
CIN: Chromosomal instability
MSI: Microsatellite instability
MOI: Multiplicity of infection
GPI: Glucose-6-phosphate isomerase
AMF: Autocrine motility factor
EMT: Epithelial–mesenchymal transition
