# Supplementary material for: Enrichment of Prevotella intermedia in human colorectal cancer and its additive effects with Fusobacterium nucleatum on the malignant transformation of colorectal adenomas
Source: J Biomed Sci. 2022 Oct 27;29:88. doi: 10.1186/s12929-022-00869-0 (PMC9615364; doi:10.1186/s12929-022-00869-0)
Supplement: Supplementary file 1 — Additional file 1. Details of data analysis of tumor-gene panel sequencing and exome sequencing. [file 12929_2022_869_MOESM1_ESM.docx]

**Additional File 1: Additional Methods**

**Analysis of next generation sequencing data**

Somatic Variant Caller (Illumina) was employed to analyze the sequencing data of the Illumina TruSight Tumor 26 Panel. Only base calls above Q20 (<1% of the estimated sequencing error) were used, and variants were called for a base position with a depth of coverage 10 or greater. The variant report was outputted as a VCF file and inputted for annotation in Illumina VariantStudio 3.0. Additional filters were applied to exclude low-quality or germline variants and to select somatic mutations unique to carcinoma samples. First, called variants marked as SB (strand bias), PB (prevalence of the variant is significantly biased between the two forward and reverse probe pools), or Low DP (sites with depth of coverage below 10) were excluded from the report. Second, variants that were observed in paired adenoma and carcinoma samples and with an alternate variant frequency of 40%—60% or close to 100% (homozygous for alternate variants) were excluded because these variants were most probably germline variants. Third, variants with an allele frequency higher than 10% in East Asian and South Asian individuals reported by 1,000 genomes were excluded to eliminate common variants. Finally, only variants with predicted biological consequences—including missense, frameshift, stop gained, stop lost, initiator codon, in-frame insertion, in-frame deletion, and splice—were included. In this study, only the somatic mutations of the *TP53* and *KRAS* genes were included for association analysis.

CASAVA1.8.2 (Illumina) was employed to analyze whole-exome sequencing data. Bcl conversion, demultiplexing, sequence alignment, and variant detection were performed in accordance with the CASAVA1.8.2 user guide. NCBI Build 37.2 was used as the reference genome for sequence alignment. Only variants located in the regions covered by the amplicons of TruSight Tumor 26 Panel were further analyzed. The called variants were filtered by Bcalls_used >= 20 and Q_snp >= 20. Annotation of the variants was performed using VarioWatch. Only variants with predicted biological consequences—including missense, frameshift, stop gained, stop lost, initiator codon, in-frame insertion, in-frame deletion, and splice—were included. Functional variants that were identified in cancerous tissues and paired non-neoplastic colon tissues were considered germline mutations and excluded from the report. Only the somatic mutations of the *TP53* and *KRAS* genes were included for association analysis.
