# Supplementary material for: Enrichment of Prevotella intermedia in human colorectal cancer and its additive effects with Fusobacterium nucleatum on the malignant transformation of colorectal adenomas
Source: J Biomed Sci. 2022 Oct 27;29:88. doi: 10.1186/s12929-022-00869-0 (PMC9615364; doi:10.1186/s12929-022-00869-0)
Supplement: Supplementary file 2 — Additional file 2. Primer and probe sequences specific to species for bacterial DNA quantification. Primer and probe sequences specific to species for bacterial DNA quantification. [file 12929_2022_869_MOESM2_ESM.docx]

**Additional file 2. Primer and probe sequences specific to species for bacterial DNA quantification.**

| Primer ID | Sequences | 5' label | Quancher | Reference |
| --- | --- | --- | --- | --- |
| PGT_F_Taq | 5'-ATCCCCAAAGCACCTGGTTT-3' |  |  | [1,2] |
| PGT_R_Taq | 5'-AGAGGCCAAGATAGTCCTGGTAA-3' |  |  |  |
| PGT_probe_Taq | 5'-CCATCCATGTCCTCATCTC-3' | FAM | MGBNFQ |  |
| FN_nusG_F | 5'-TGGTGTCATTCTTCCAAAAATATCA-3' |  |  | [1] |
| FN_nusG_R | 5'-AGATCAAGAAGGACAAGTTGCTGAA-3' |  |  |  |
| FN_nusG_P | 5'-ACTTTAACTCTACCATGTTCA-3' | FAM | MGBNFQ | |
| PI_F_Taq | 5'-CCACATATGGCATCTGACGTG-3' |  |  |  |
| PI_nR_Taq | 5'-CTGATCGTAGCCTTGGTGGG-3' |  |  |  |
| PI_probe_Taq | 5'-ACCAAAGATTCATCGGTGGAGGATGGG-3' | FAM | QSY |  |
| BF_bft_F | 5'-GGATAAGCGTACTAAAATACAGCTGGAT-3' |  |  | [3] |
| BF_bft_R | 5'-CTGCGAACTCATCTCCCAGTATAAA-3' |  |  |  |
| BF_bft_P | 5'-CAGACGGACATTCTC-3' | FAM | MGBNFQ | |

**References**

1. Yamamura K., Baba Y., Miyake K., Nakamura K., Shigaki H., Mima K., Kurashige J., Ishimoto T., Iwatsuki M., Sakamoto Y., Yamashita Y., Yoshida N., Watanabe M. and Baba H. Fusobacterium nucleatum in gastroenterological cancer: Evaluation of measurement methods using quantitative polymerase chain reaction and a literature review. Oncol Lett 14(6):6373-6378, 2017.

2. Castellarin M., Warren R.L., Freeman J.D., Dreolini L., Krzywinski M., Strauss J., Barnes R., Watson P., Allen-Vercoe E., Moore R.A. and Holt R.A. Fusobacterium nucleatum infection is prevalent in human colorectal carcinoma. Genome Res 22(2):299-306, 2012.

3. Purcell R.V., Pearson J., Frizelle F.A. and Keenan J.I. Comparison of standard, quantitative and digital PCR in the detection of enterotoxigenic Bacteroides fragilis. Sci Rep 6:34554, 2016.
