# Supplementary material for: Enrichment of Prevotella intermedia in human colorectal cancer and its additive effects with Fusobacterium nucleatum on the malignant transformation of colorectal adenomas
Source: J Biomed Sci. 2022 Oct 27;29:88. doi: 10.1186/s12929-022-00869-0 (PMC9615364; doi:10.1186/s12929-022-00869-0)

Figure S1 No dose-dependent effect of *Prevotella intermedia* on enhancing CRC cell migration and invasion

CRC cells were treated with *Prevotella intermedia* at MOI of 100:1 or 250:1. Relative percentage of migrated (A – C) and invaded (D – F) cells are presented as mean ± SD. The data are average from three independent experiments. ****: P-value < 0.0001; ns: not significant.

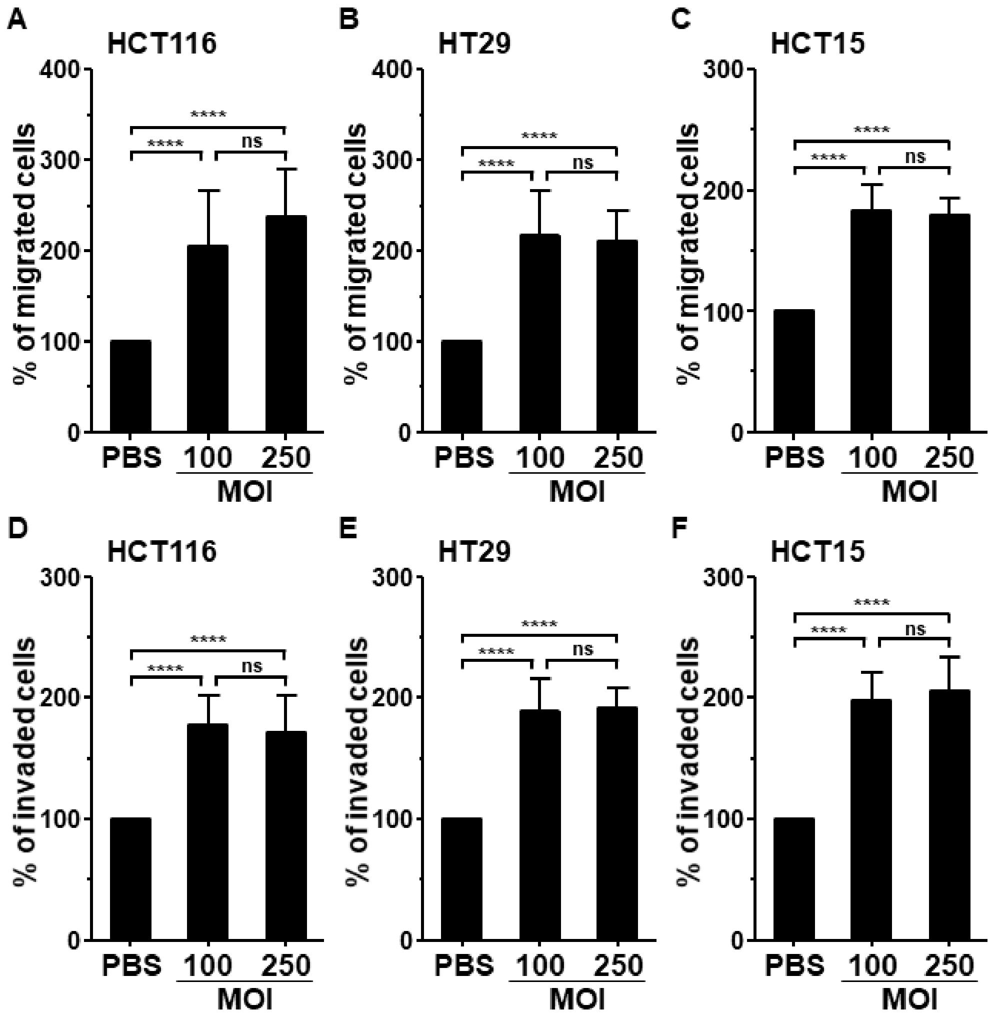

Supplement: Supplementary file 6 — Additional file 6: Figure S1. Migration and invasion of CRC cells incubated with Prevotella intermedia at various MOIs. [file 12929_2022_869_MOESM6_ESM.pdf]
