# Supplementary material for: Enrichment of Prevotella intermedia in human colorectal cancer and its additive effects with Fusobacterium nucleatum on the malignant transformation of colorectal adenomas
Source: J Biomed Sci. 2022 Oct 27;29:88. doi: 10.1186/s12929-022-00869-0 (PMC9615364; doi:10.1186/s12929-022-00869-0)

**Figure S2** Gel electrophoresis of conditioned medium from *Prevotella intermedia* with or without proteinase K treatment.

Conditioned medium from *Prevotella intermedia* treated with proteinase K was separated by SDS-PAGE and visualized with silver stain. Conditioned medium without treatment was also presented and medium alone served as a negative control.

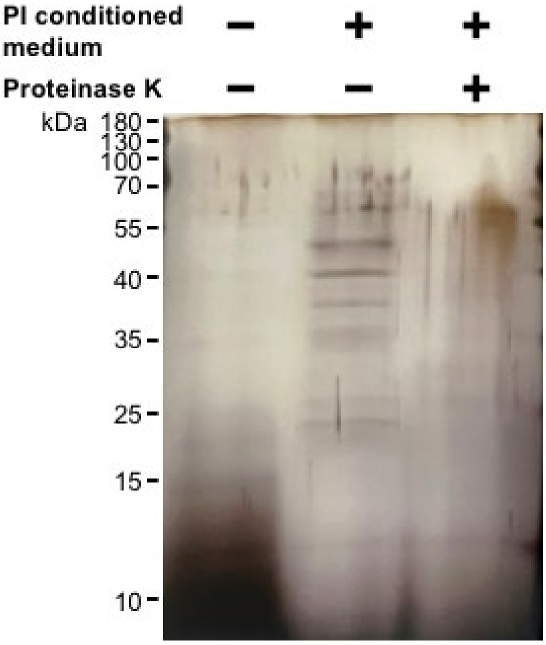

Supplement: Supplementary file 7 — Additional file 7: Figure S2. Silver staining of conditioned medium collected from Prevotella intermedia. [file 12929_2022_869_MOESM7_ESM.pdf]
