# Supplementary material for: Enrichment of Prevotella intermedia in human colorectal cancer and its additive effects with Fusobacterium nucleatum on the malignant transformation of colorectal adenomas
Source: J Biomed Sci. 2022 Oct 27;29:88. doi: 10.1186/s12929-022-00869-0 (PMC9615364; doi:10.1186/s12929-022-00869-0)

Figure S3 Dose effect of *Fusobacterium nucleatum* on CRC cell growth.

HCT116 cells were treated with *Fusobacterium nucleatum* at an MOI of 250, 500, or 1000 on day 0. Cell growth curves were measured using a hemocytometer. Cells treated with PBS served as the controls. *: P value < 0.05; **: P value < 0.01.

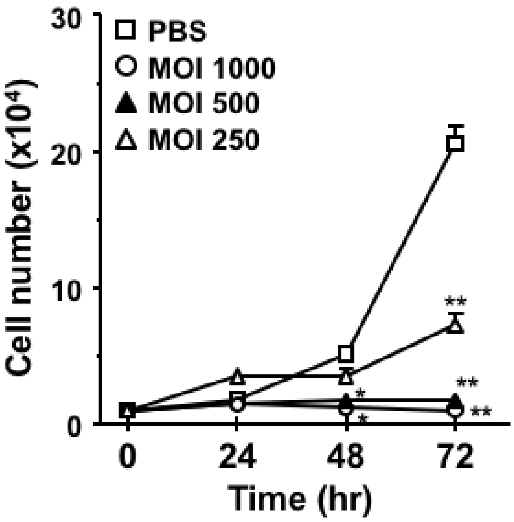

Supplement: Supplementary file 8 — Additional file 8: Figure S3. Growth of CRC cells incubated with Prevotella intermedia at various MOIs. [file 12929_2022_869_MOESM8_ESM.pdf]
